# Supplementary material for: Predicting mammalian hosts in which novel coronaviruses can be generated
Source: Nat Commun. 2021 Feb 16;12:780. doi: 10.1038/s41467-021-21034-5 (PMC7887240; doi:10.1038/s41467-021-21034-5)
Supplement: Supplementary file 1 — Supplementary Information [file 41467_2021_21034_MOESM1_ESM.pdf]

Predicting mammalian hosts in which novel coronaviruses can be generated

Supplementary Information

Maya Wardeh*, Matthew Baylis, Marcus SC Blagrove*

*Correspondence to: maya.wardeh@liverpool.ac.uk. marcus.blagrove@liverpool.ac.uk.

Supplementary Note 1 – From mammalian phylogenetic, ecological, and geo-spatial traits to mammalian similarity

Phylogeny: Mammalian phylogenetic distance has been linked to sharing of viruses[1–3]. We calculated pair wise phylogenetic similarity between each mammal-mammal pair based on phylogenetic distances extracted from a recent mammalian supertree[4].

Ecological traits**:** We compiled data on morphological and life-history traits, diet and habitat for our mammal species from online databases and literature[1,5–9]. We selected the following traits for their known correlation with host-pathogen associations, and their wide availability: Body mass (g), maximum age (months), proxied key features of metabolism and adaption to environment; activity cycle, and migration[6] presented key aspects of mammalian behaviour. We utilised the following reproductive traits: age at sexual maturity (days), gestation period length (days), litters per year, litter size and weaning age (days). Reproductive traits could be viewed as proxies to within-host virus-dynamics and therefore may influence the viruses harboured by the host.

We incorporated the above traits to calculate traits-based pairwise distance between each two mammalian species. We based these distance calculations a generalised form of Gower's distance matrices[10,11]. We then transformed these distances into similarities (similarity between two mammals = 1- normalised distance).

Mammals utilising similar habitats might encounter similar coronaviruses and this in turn would increase the chances of being infected with these coronaviruses. We therefore incorporated habitat utilisation[8] as multiple binary indicators of whether a species uses one or more of 14 natural and artificial habitats. We transformed these habitat utilisations into similarity matrix following same procedure as above.

In addition to habitat, similar diet preference, expressed in terms of proportional use of 10 diet categories[7], could potentially associate with similar viral assemblage. We transformed these diet categories into a similarity matrix as per above.

The above steps we resulted in the following pair-wise ecological similarities between each pair of mammals in our study:

1. Life-history and reproductive traits similarity
2. Habitat utilisation similarity
3. Diet similarity

Geospatial traits**:** The geographical distribution of mammalian species influences the coronaviruses with which they might come into contact. Geographical spread correlates with other factors such as climate, natural environment, and agricultural practices (including potential contact with livestock). Climate has been shown to influence a number of human and domestic mammal pathogens (including viruses)[12–14]. Other geographical factors such as biodiversity (species richness), land cover type, agriculture and farming practices, urbanisation and human population have been found to influence certain categories of host-pathogen associations[15,16].

Species-presence maps: We obtained species-presence maps for majority of our mammalian species from IUCN[8]. We extrapolated livestock (including horses – *Equus caballus*) species-presence maps from most recent global distribution maps[17]. Finally, we inferred presence-maps for three domesticated species - dogs (*Canis lupus familiaris*), cats (*Felis catus)* and guinea pigs (*Cavia porcellus*) from Gridded population of the world maps[18], by assuming they co-exist with humans where there is sufficient human populations (n>100). We used the same gridded population maps to extrapolate human species-presence map (n>0).

Presence overlap: we intersected the above curated maps using the R Package raster to compute whether the presence of any two mammalian species in our input overlapped (binary, 1=yes, 0=no), and to calculate the area of this overlap (in km^2).

Vectorised geospatial features: We supplemented species-presence maps with grids expressing climate[19], mammalian diversity[20], human population[18], land cover (including urbanisation)[21], agriculture[21,22], and distribution of livestock[17]. This allowed to generate the following geospatial feature vectors for each mammalian species (Supplementary Figure 2):

1. Climate: we expressed climate in two vectorised features as follows:
    a. Mean temperature: we computed mean of monthly temperatures recorded in each grid (Supplementary Table 1) in the species-presence area, averaged between years: 1900-2010[19]. WE transformed this gridded temperature in to an 11-points quantile vector

representing the probabilities: 5%, 10%, 20%, 30%, 40%, 50%, 60%, 70%, 80%, 90%, and 95%.

b. Mean precipitation: Sum of monthly rainfall (precipitation) recorded in each grid in the species-presence area, averaged between years: 1900-2010[19]. Gridded precipitation was transformed into quantile vector as above.

2. Natural land-cover type (not directly associated with humans): we computed vectorised features (above) for each of the land-cover types in this category (Supplementary Table 1).
3. Agricultural land-cover type (including land-cover associated with humans e.g., managed vegetation) and farming practices (expressed in number of domesticated livestock and poultry in the species presence area) were also quantified into vectorised features as above.
4. Urbanisation and human population[18] vectorised features were computed form species-presence maps.
5. Mammalian diversity[20] in the species presence area was transformed into vectorised features.

We transformed the above vectors into pair-wise mammal-mammal similarity matrices by calculating cosine similarity between the vectorised features (as per the viral similarities calculated in manuscript).

Similarity network fusion (SNF): We applied similarity network fusion (SNF)[32] to integrate the following similarity matrices calculated above in order to reduce our mammalian feature space:

1. Climate similarities: temperature and precipitation similarities were integrated using SNF.
2. Geo-spatial traits: the 7 similarities derived from natural land-cover type (Supplementary Table 1), the 11 similarities derived from agricultural land-cove type and farming (livestock and poultry); the 2 similarities based on urbanisation and human population, and the mammalian diversity similarity were integrated using SNF.

Category	layers(s)/geo-attributes	Source	Res	Reason
**(Natural) Land-cover**	Evergreen/deciduous needle-leaf trees (%)	EarthEnv[21]	0°0′30″	Type of land cover has been associated with distribution of various mammals[23]. It potentially increases chances of contact between mammalian reservoirs of different viruses.
	Evergreen broad-leaf trees (%)			
	Deciduous broad-leaf trees (%)			
	Mixed/other trees (%)			
	Shrubs (%)			
	Herbaceous vegetation (%)			
	Barren land (%)			
**Agriculture & farming**	Managed/Cultivated Vegetation (%)	EarthEnv[21]	0°0′30″	
	Regularly flooded vegetation (%)			
	Cropland (%)	HYDE[24]	0°5′	
	Pasture (%)			
	Cattle (head count)	Global livestock[17]	0.0833°	Livestock farming is linked to cross-species transmission of number of viruses[25].
	Sheep (head count)			
	Buffalo (head count)			
	Pigs (head count)			
	Horses (head count)			
	Chicken (head count)			
	Duck (head count)			
**Human**	Human population	SEDAC[18]	0°5′	Urbanisation and human population density have been shown to be drivers of viral spill-over through wildlife-domestic-human interface[16,26,27].
	Urban land (%)	EarthEnv[21]	0°0′30″	
**Climate**	Mean temperature	CRUTS3[19]	0°5′	Climate could potentially influence the spread and emergence of viruses[12–14].
	Mean precipitation			
**Mammalian diversity**	Number of different mammalian species in a grid cell.	SEDAC[20]	0°5′	Mammalian species present in mammal rich areas might be exposed to diverse viruses[28–31].

Supplementary Table 1. List of geographical predictor layers integrated within our framework.

Supplementary Note 2 – DeepWalk

We adopted DeepWalk[33] to compute vectorised representations for our coronaviruses and hosts from the network connecting them. DeepWalk uses truncated random walks to get latent topological information of the network and obtains the vector representation of its nodes (in our case coronaviruses, and their hosts) by maximising the probability of reaching a next node (i.e., probability of a virus-host association) given the previous nodes in these walks.

DeepWalk comprises three steps (Supplementary Figure 3):

1. Sampling*:* For each node n_i (virus or host) in our network, DeepWalk conducts γ random walks with length t starting from n_i.
2. Training skip-gram*:* by treating walks as the equivalent of sentences, DeepWalk updates the node representation using the skip-gram algorithm[34] for each walk (Supplementary Figure 3). Here, skip-gram is used to maximise the cooccurrence probability among nodes which appear within a window w using an independent assumption as follows:

$$\Pr\left(\{n_{i-w}, \ldots, n_{i+w}\}\backslash n_i | \phi(n_i)\right) = \prod_{j=i-w, j\neq i}^{i+w} \Pr\left(n_i | \phi(n_i)\right) \quad \text{(S1)}$$

where Φ denotes the latent topological representation associated with every vertex n_i. Φ is represented by an $|\mathbf{N}| \times d$ matrix, where $|\mathbf{N}|$ is the cardinality of node set **N**, and d is the dimension of the node vector.

$\Pr\left(n_i|\phi(n_i)\right)$ is approximated with Hierarchial Softmax[35] by assigning the nodes to the leaves of a Huffman tree, and $\Pr\left(n_i|\phi(n_i)\right)$ can be computed as:

$$\Pr\left(n_i|\phi(n_i)\right) = \prod_{l=1}^{\lceil \log|\mathbf{N}| \rceil} \frac{1}{\left(1 + e^{-\phi(n_i)\psi(b_l)}\right)} \quad \text{(S2)}$$

where $b_l \in \left(b_0, b_{21}, .., b_{\lceil \log|\mathbf{N}| \rceil}\right)$, and $\psi(b_l)$ is the representation assigned to the parent of node b_l. $\left(b_0, b_{21}, .., b_{\lceil \log|\mathbf{N}| \rceil}\right)$ is a sequence of tree nodes to identify the node n_i, such that b_0 is the root of this tree and $b_{\lceil \log|\mathbf{N}| \rceil} = n_i$.

3. Computing embeddings: After completing the above step, the latent topological representation of nodes in the network is the output of a hidden layer of the network.

DeepWalk performs its walks at random which means that embeddings do not preserve the local neighbourhood of the nodes well. However, other components of our pipeline, capture these local information from the virus and the mammalian perspectives.

Supplementary Note 3 – Changes in network structure with addition of predicted links

Definitions

$\mathbf{L}$	Realised links in the network. For our original network, this number equals knowns associations between our Coronaviruses (CoVs) and their mammalian hosts. In our predicted networks $\mathbf{L}$ contains known and predicted links at the given probability cut-off.
$\mathbf{M}$	Mammalian species in the network.
$\mathbf{V}$	CoVs in the network
$\mathbf{A}$	Adjacency matrix of dimensions $\lvert\mathbf{V}\rvert \times \lvert\mathbf{M}\rvert$, such that for each $v_i \in \mathbf{V}$ and $m_j \in \mathbf{M}$, $a_{ij} = 1$ if an association exists (or is predicted at given cut-off) between the coronavirus and the mammal, and 0 otherwise.
$\mathbf{A}_i$	Mammals associated with coronavirus $v_i \in \mathbf{V}$. Such that, $\mathbf{A}_i = \sum_{j=1}^{\lvert M\rvert} \mathbf{A}a_{ij}$. Corresponds to degree centrality (from the viral perspective).
$\mathbf{A}_j$	CoVs associated with mammal $m_j \in \mathbf{M}$. Such that, $\mathbf{A}_j = \sum_{i=1}^{\lvert\mathbf{V}\rvert} \mathbf{A}a_{ij}$. Corresponds to degree centrality (from the viral perspective).

Given the above definitions we computed the following structural properties at the level of the whole network, and the group (i.e., CoVs or mammals).

- Mean degree – $D = \frac{\lvert\mathbf{L}\rvert}{\lvert\mathbf{V}\rvert+\lvert\mathbf{M}\rvert}$: mean number of associations per CoV and mammal.
- Connectance – $C = \frac{\lvert\mathbf{L}\rvert}{\lvert\mathbf{V}\rvert\times\lvert\mathbf{M}\rvert}$: realised proportion of possible associations (links). Deterministically increases with addition of new associations.
- Cluster coefficient: mean per-node (CoV or mammal) connectance, equals to mean, across all CoVs and mammals, of the number of realised associations (i.e., known and/or predicted) divided by the number of possible links for each node (CoV or mammal).
- Cluster coefficient (CoVs): mean, across all CoVs, of the number of realised associations divided by the number of possible links for each CoV.
- Cluster coefficient (mammals): mean, across all mammals, of the number of realised associations divided by the number of possible links for each mammal.
- Mean number of shared partners (CoVs): simple measure of co-occurrence, captures mean number of shared hosts of CoVs.
- Mean number of shared partners (mammals): simple measure of co-occurrence. Capture mean number of shared CoVs between mammalian hosts.
- Togetherness (CoVs): measures the tendency of CoVs to be found in the same mammalian hosts. A high level (1) of togetherness (CoVs) suggests that the availability of a common trait or

characteristics in these mammals (e.g. common receptor) might important in ability of CoVs to infect/association with them, whereas lower level (0) indicates the opposite[36,37].

- Togetherness (mammals): captures the tendency of mammalian species to shares CoVs. High values (max=1) of togetherness (mammals) suggest that similarities between mammalian species (e.g., habitat or diet requirements) might be more important driver of sharing of CoVs (community structure) than competition. Smaller values (min=0) indicate the opposite[36,37].
- C-Score - Checkerboard score[38] (averaged across all nodes in level, i.e. CoVs or mammals): this score measures non-independence in interaction patterns across the network.
    - Larger values of C-Score (CoVs) indicates mammalian communities with little or no overlap in shared CoVs.
    - Larger values of C-Score (mammals) suggest CoVs communities with little or no overlap in host preferences (e.g., tendencies of CoVs to be shared amongst certain host communities, defined, for example by phylogeny or geographical distribution).
- V-ratio – variance-to-mean ratio: Larger values of V-ratio (CoVs) indicate a more skewed host range of our CoVs. Larger values of V-ratio (mammals) indicate a more skewed richness of CoVs in our mammalian hosts.
- Nestedness - NODF (nestedness metric based on overlap and decreasing fill)[39]: nestedness captures the tendency of specialists (e.g., CoVs with few hosts) to interact with (e.g., infects) subsets of mammals with which generalists (e.g., CoVs with many hosts) interact. It has been linked to network stability and functionality[40,41].
- Niche overlap (CoVs): mean similarity in interaction pattern between CoVs (in relation to mammalian species). Here we calculate this similarity via Horn's index (default implementation in the R package bipartite[42]). Niche overlap ranges from 0 (indicating no common pattern in how CoVs associate with mammalian species, to 1 indicating perfect overlap).
- Niche overlap (mammals): mean similarity in interaction pattern between mammals (in relation to CoVs), see above.
- Robustness – area below the "secondary extinction" curve.
    - Robustness (CoVs): CoVs are deleted at random, and area under the "second extinction" curve is calculated[42].
    - Robustness (mammals): mammalian species are deleted at random, and area under the "second extinction" curve is calculated[42]. Large values of robustness (max = 1) indicate a curve that decreases very mildly until the point at which almost all mammalian species are eliminated. This suggests a very robust system in which, for instance, circulation of CoVs continues even if large fraction of mammalian host species is eliminated. On the other hand

low values of robustness correspond to a curve that decreases abruptly as soon as any host species is lost. This is consistent with a fragile system in which, for instance, even if a very small fraction of the mammalian host is eliminated, most of CoVs lose their preferred hosts and drop from network.

Metric	original	cut-off>=0.9821	cut-off>0.75	cut-off>0.5
**Mammalian diversity per virus**	0.057	0.093 (0.085 - 0.51) / 1.632-fold (1.491 - 8.947)	0.543 (0.131 - 0.548) / 9.526-fold (2.298 - 9.614)	0.519 (0.331 - 0.595) / 9.11-fold (5.81 - 10.44)
**Viral diversity per mammal**	0.241	0.501 (0.27 - 0.73) / 2.079-fold (1.12 - 3.029)	0.731 (0.666 - 0.729) / 3.033-fold (2.763 - 3.025)	0.73 (0.73 - 0.72) / 3.03-fold (3.03 - 2.99)
**Mean degree (associations per CoV or mammal)**	1.055	2.052 (1.306 - 9.178) / 1.945-fold (1.238 - 8.699)	6.785 (3.433 - 11.41) / 6.431-fold (3.254 - 10.815)	9.398 (5.718 - 13.683) / 8.91-fold (5.42 - 12.97)
**Connectance**	0.011	0.017 (0.011 - 0.073) / 1.524-fold (1.022 - 6.626)	0.054 (0.027 - 0.091) / 4.909-fold (2.455 - 8.273)	0.075 (0.045 - 0.108) / 6.82-fold (4.09 - 9.82)
**Cluster coefficient**	0.005	0.004 (0.004 - 0.017) / 0.712-fold (0.8 - 3.333)	0.041 (0.023 - 0.074) / 8.2-fold (4.6 - 14.8)	0.06 (0.037 - 0.088) / 12-fold (7.4 - 17.6)
**Cluster coefficient (CoVs)**	0.041	0.044 (0.037 - 0.123) / 1.078-fold (0.912 - 2.995)	0.096 (0.056 - 0.153) / 2.341-fold (1.366 - 3.732)	0.125 (0.08 - 0.182) / 3.05-fold (1.95 - 4.44)
**Cluster coefficient (mammals)**	0.096	0.205 (0.159 - 0.529) / 2.138-fold (1.652 - 5.516)	0.483 (0.318 - 0.539) / 5.031-fold (3.313 - 5.615)	0.53 (0.447 - 0.549) / 5.52-fold (4.66 - 5.72)
**Nestedness (NODF)**	6.065	24.599 (12.979 - 61.513) / 4.056-fold (2.14 - 10.142)	56.856 (38.165 - 65.653) / 9.374-fold (6.293 - 10.825)	61.705 (51.99 - 68.843) / 10.17-fold (8.57 - 11.35)
**Mean number of shared partners (CoVs)**	0.207	0.736 (0.379 - 8.35) / 3.556-fold (1.83 - 40.338)	5.627 (1.876 - 10.567) / 27.184-fold (9.063 - 51.048)	8.555 (4.39 - 12.849) / 41.33-fold (21.21 - 62.07)
**Mean number of shared partners (mammals)**	0.072	0.187 (0.093 - 2.596) / 2.601-fold (1.286 - 36.059)	1.488 (0.419 - 4.056) / 20.667-fold (5.819 - 56.333)	2.711 (1.026 - 5.855) / 37.65-fold (14.25 - 81.32)
**Niche overlap (CoVs)**	0.13	0.233 (0.222 - 0.564) / 1.79-fold (1.708 - 4.335)	0.505 (0.318 - 0.579) / 3.885-fold (2.446 - 4.454)	0.564 (0.459 - 0.589) / 4.34-fold (3.53 - 4.53)
**Niche overlap (mammals)**	0.053	0.056 (0.053 - 0.185) / 1.06-fold (1 - 3.488)	0.149 (0.065 - 0.225) / 2.811-fold (1.226 - 4.245)	0.187 (0.11 - 0.254) / 3.53-fold (2.08 - 4.79)
**Togetherness (CoVs)**	0.007	0.02 (0.012 - 0.137) / 2.828-fold (1.766 - 19.572)	0.094 (0.042 - 0.175) / 13.429-fold (6 - 25)	0.14 (0.078 - 0.195) / 20-fold (11.14 - 27.86)
**Togetherness (mammals)**	0.002	0.002 (0.001 - 0.017) / 1.172-fold (0.693 - 8.334)	0.01 (0.004 - 0.026) / 5-fold (2 - 13)	0.017 (0.007 - 0.036) / 8.5-fold (3.5 - 18)
**C-score (CoVs)**	0.811	0.56 (0.666 - 0.125) / 0.69-fold (0.821 - 0.154)	0.165 (0.381 - 0.117) / 0.203-fold (0.47 - 0.144)	0.124 (0.213 - 0.104) / 0.15-fold (0.26 - 0.13)
**C-score (mammals)**	0.931	0.877 (0.92 - 0.53) / 0.942-fold (0.988 - 0.569)	0.61 (0.81 - 0.437) / 0.655-fold (0.87 - 0.469)	0.531 (0.689 - 0.378) / 0.57-fold (0.74 - 0.41)
**V-ratio (CoVs)**	7.207	7.489 (6.744 - 23.017) / 1.039-fold (0.936 - 3.194)	17.781 (9.083 - 29.415) / 2.467-fold (1.26 - 4.081)	23.29 (13.412 - 35.652) / 3.23-fold (1.86 - 4.95)
**V-ratio (mammals)**	16.459	55.402 (38.269 - 156.159) / 3.366-fold (2.325 - 9.488)	142.419 (90.995 - 158.811) / 8.653-fold (5.529 - 9.649)	156.054 (131.029 - 163.227) / 9.48-fold (7.96 - 9.92)
**Robustness (CoVs)**	0.538	0.559 (0.547 - 0.808) / 1.039-fold (1.017 - 1.502)	0.724 (0.578 - 0.856) / 1.346-fold (1.074 - 1.591)	0.818 (0.671 - 0.896) / 1.52-fold (1.25 - 1.67)
**Robustness (mammals)**	0.586	0.69 (0.609 - 0.926) / 1.178-fold (1.04 - 1.58)	0.896 (0.785 - 0.941) / 1.529-fold (1.34 - 1.606)	0.926 (0.878 - 0.948) / 1.58-fold (1.5 - 1.62)

Supplementary Table 2. Network measures calculated for four bipartite networks (as presented in Figure 3): original network (3A), predicted network at probability cutoffs: ≥0.9821 (3B), >0.75 (3C), and >0.5 (3D), respectively. Values in bracket SD from ensemble mean.

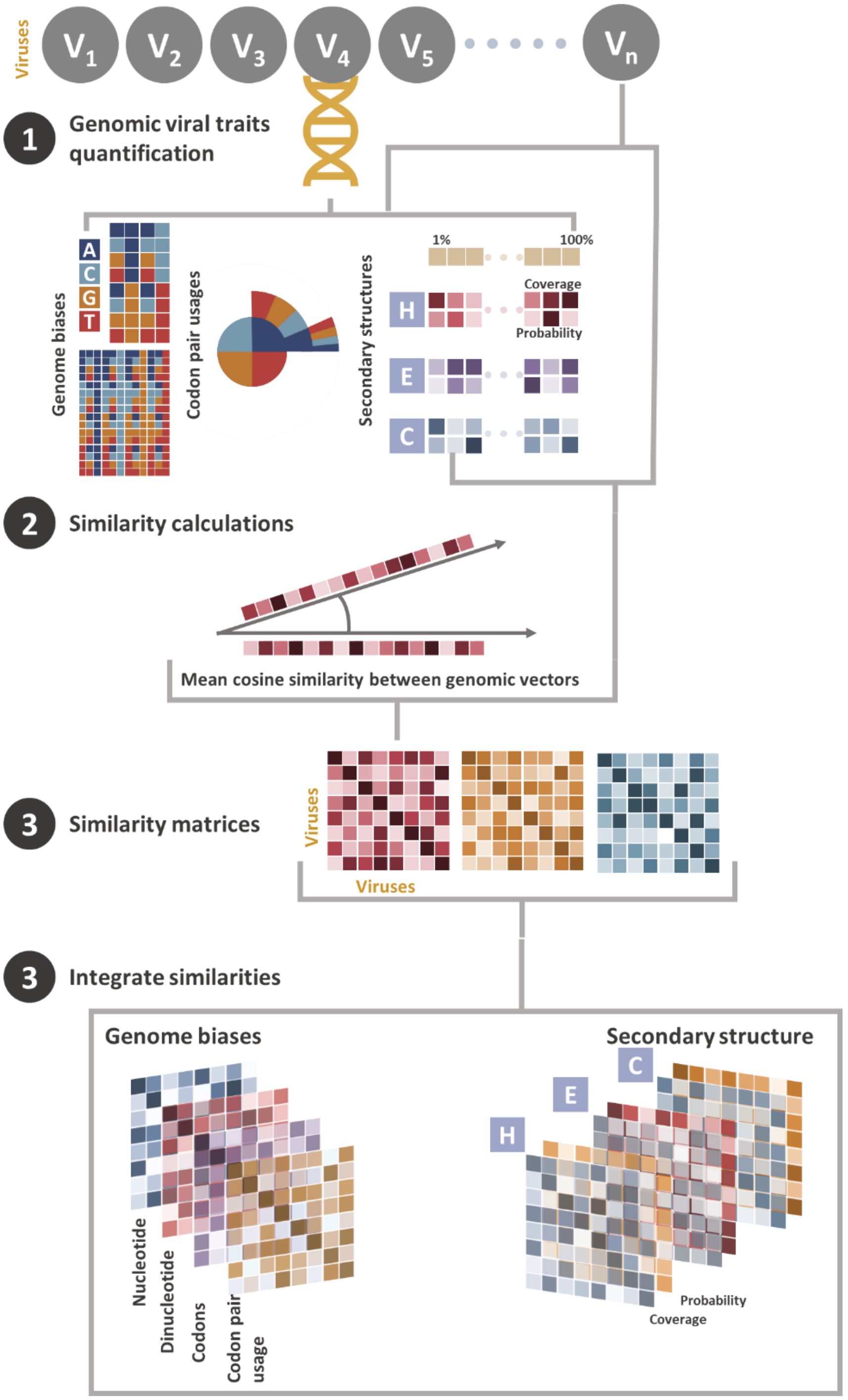

Supplementary Figure 1. Computing genomic-features similarity matrices of coronaviruses. First genomic traits were extracted from complete genome sequences of coronaviruses– step 1. Then the vector representations of each trait were used to compute cosine similarity between two genomes for the trait (e.g., dinucleotide biases) and the mean was taken when computing similarity between two coronaviruses represented by one or more genome each – step 2. The resulting similarity matrices were integrated using SNF[32] to generate two integrated similarity matrices: genome biases similarity and secondary structure similarity.

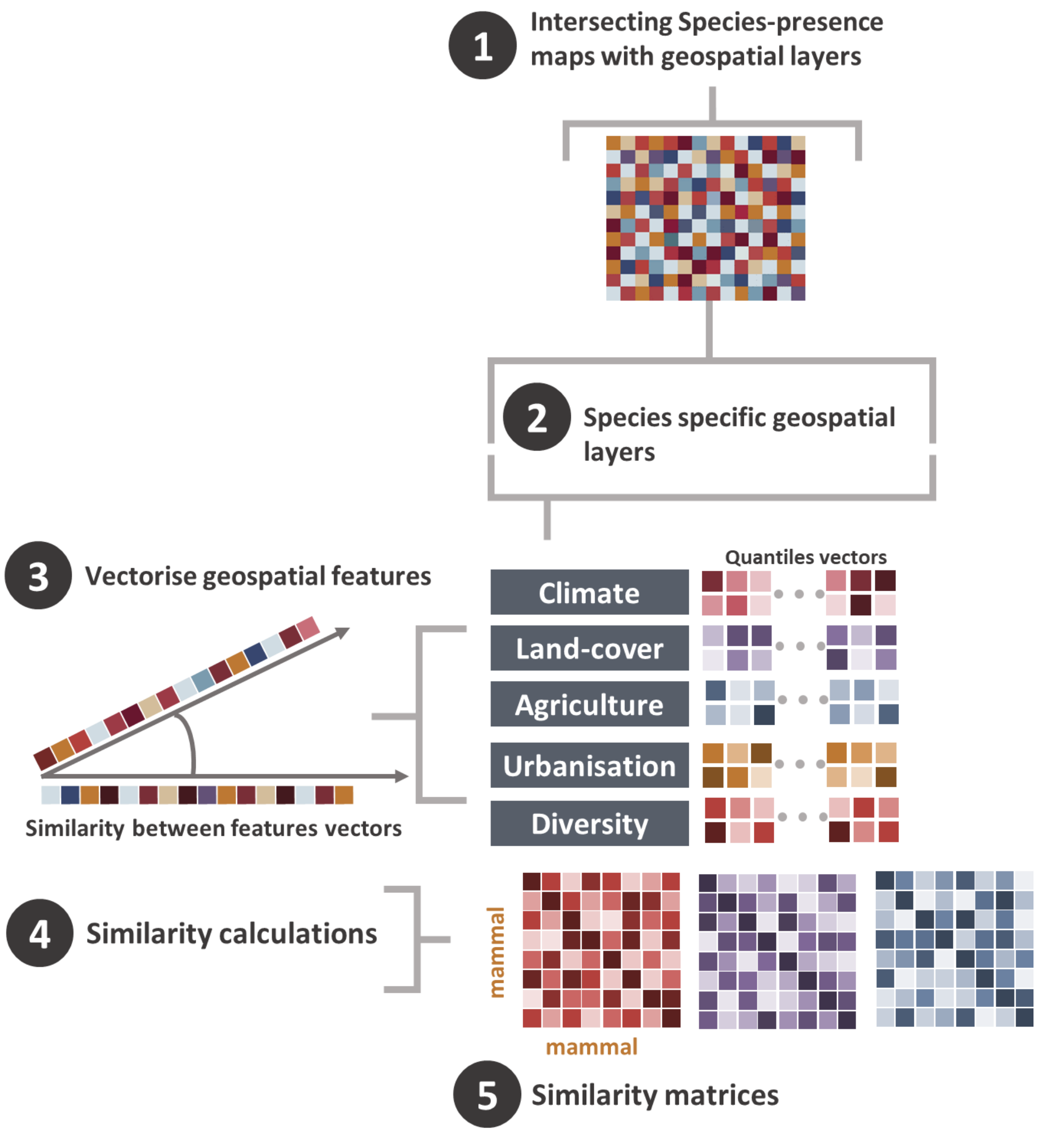

Supplementary Figure 2. Vectorised geospatial features extraction. Mammalian species-presence maps were first extracted from our sources[8,17,18]; these maps were then intersected with our geospatial layers (Supplementary Table 1) – step1. This enabled us to derive quantile vectors of our geospatial attributes (Supplementary Table 1) for majority of our mammalian species – step2, which we then transformed into vectors expressing quantile distribution of these attributes in the species presence area – step 3. Finally, we computed cosine similarity between these vectors to generate a pair-wise similarity matrix (between mammalian species) for each of our geospatial attributes – step 4.

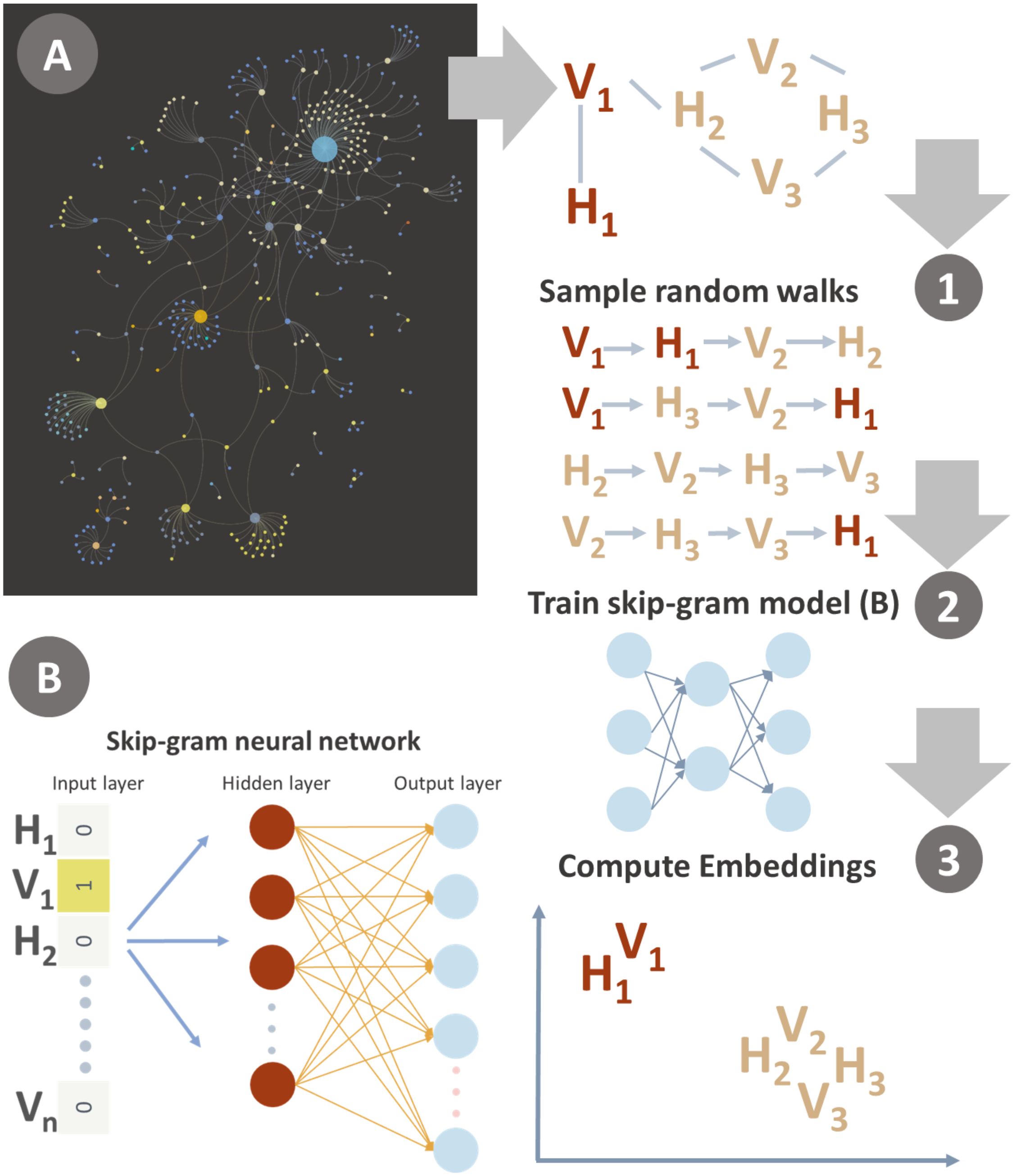

Supplementary Figure 3. Network embeddings using DeepWalk. A – DeepWalk process. B Skip-gram neural network: the network is composed of input layer, hidden layer(s) and output layer. The network accepts one-hot encoded vector. One-hot encoding is a vector with length same = number of nodes in network (or words in dictionary in the original implementation). The hidden layer has no activation function, its output presents an embedding of the node. The output layer is a *SoftMax* classifier that predicts neighborhoud nodes. V represent viruses and H represent mammalian hosts in this figure.

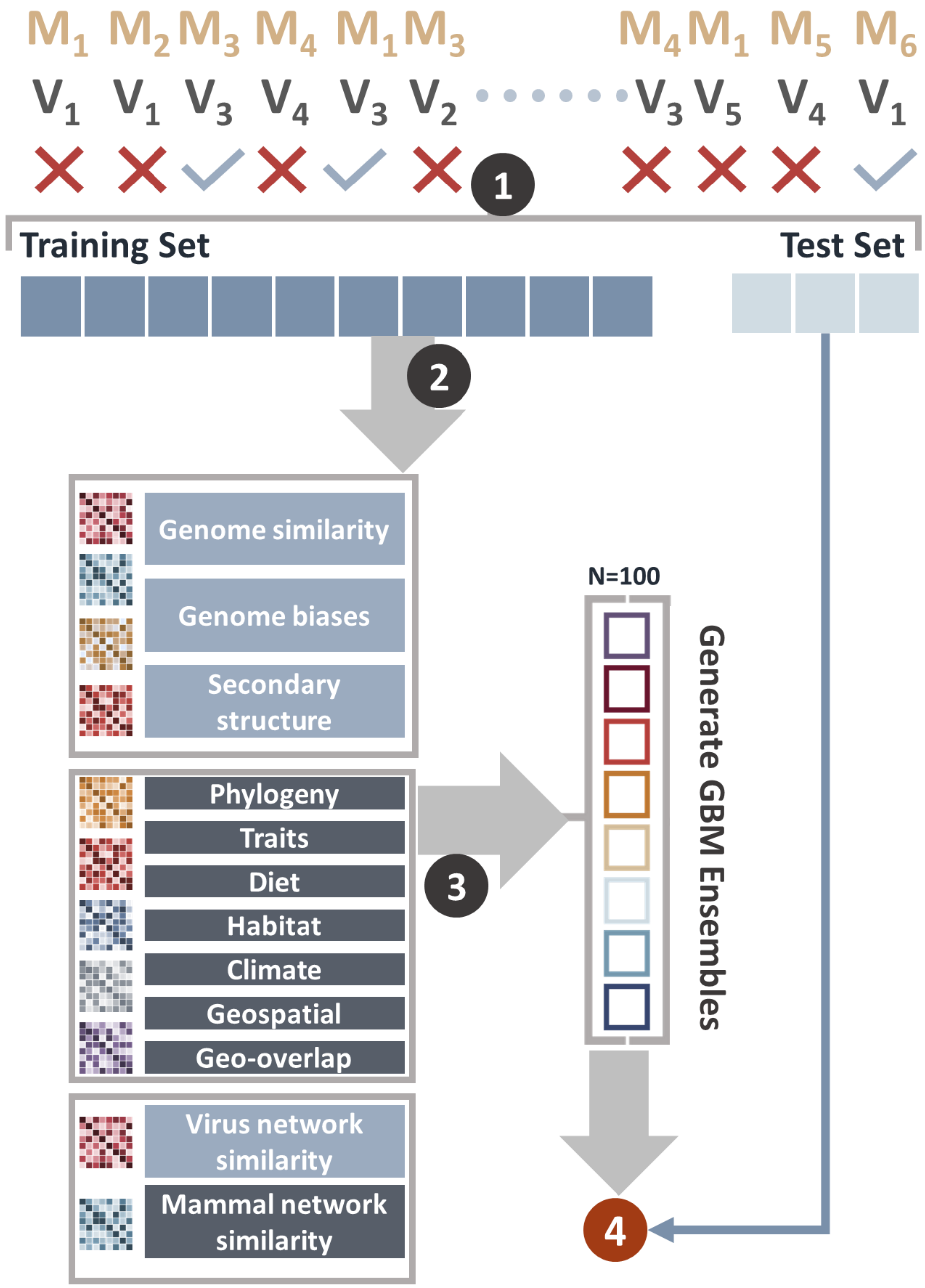

Supplementary Figure 4. Visualisation of training and performance assessment (20 repeats). Step 1 – observed associations between CoVs and their hosts is split into training set comprising 85% of all observed associations – and test-set comprising 15% of these associations. Step 2 – training set is used to generate similarity learners in three categories: coronaviruses, mammalian hosts and networks. Network perspective learners are recomputed for each test run (from the reduced network). Step 3 – GBM is applied to generate meta-ensembles integrating the meta-learners. The ensembles comprise 100 replicate models trained with balanced samples drawn from the combined learners results. Step 4 – the model performance of the GBM ensemble is assessed by taking the mean probability of the 100 replicate models as applied to the test set. V represent viruses and H represent mammalian hosts in this figure.

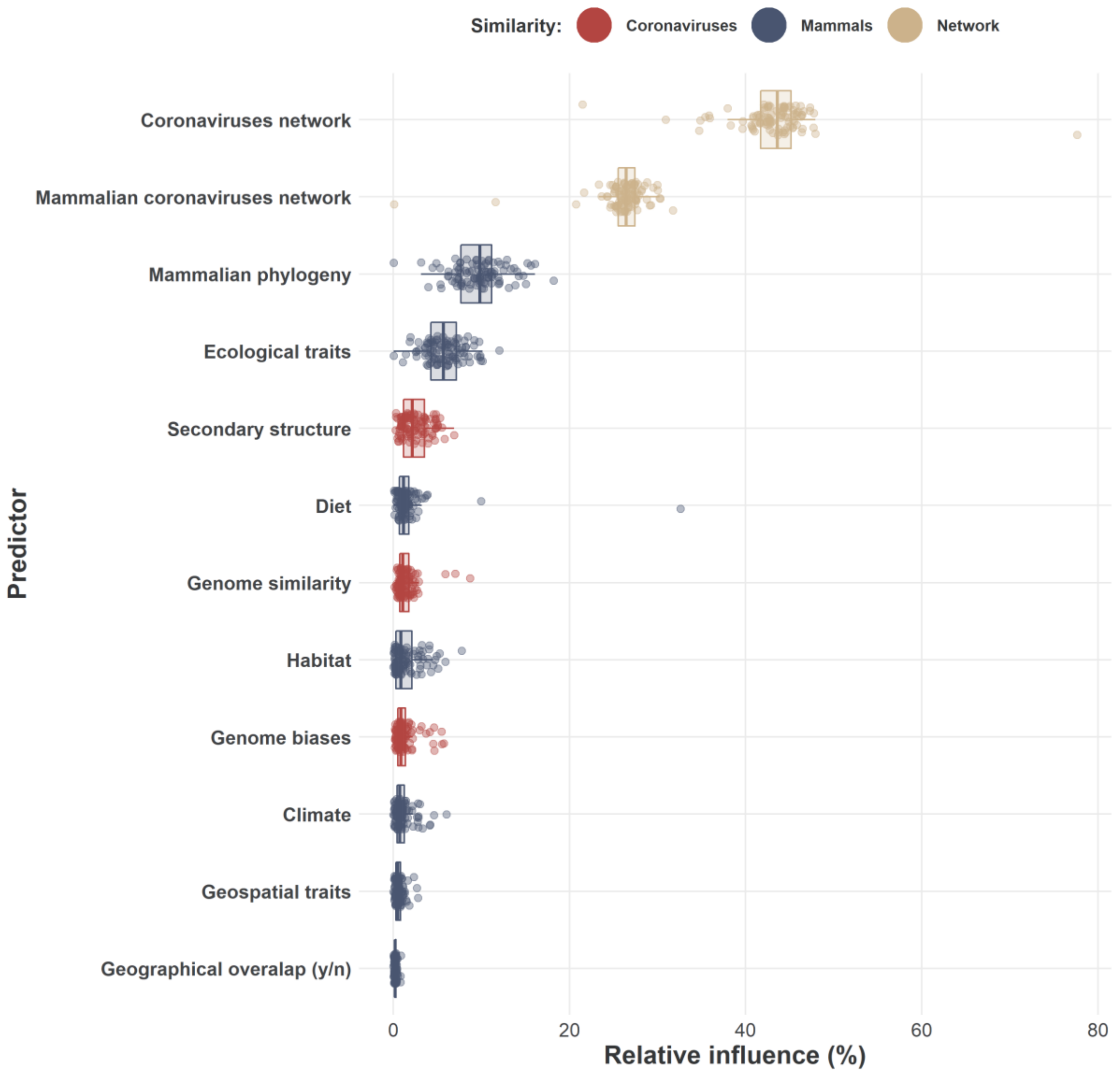

Supplementary Figure 5. Relative influence (variable importance) of the included learners to the final GBM ensembles (trained with all available associations). Boxplots indicate median (center), the 25th and 75th percentiles (bounds of box) and inter quantile range (whiskers). Points represent the relative influence of the learner in individual runs. (100 runs in total). Boxplots and points are coloured by learner's point of view (perspective): network, mammalian or viral. Relative influence of each learner to each run (n=100) of our GBM meta-ensemble was obtained using varImp function in R package caret. Learners are ordered by their median relative influence over the 100 runs.

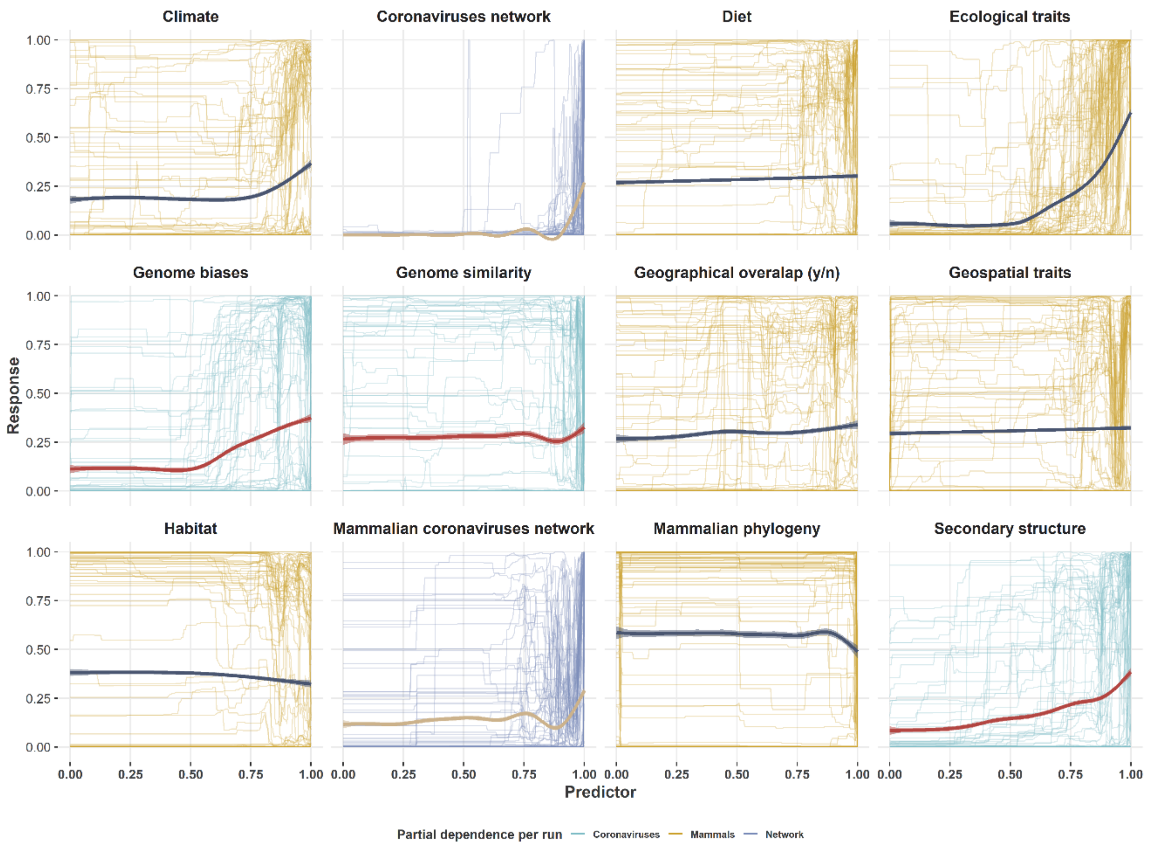

Supplementary Figure 6. Partial dependence plots showing the influence on coronavirus-mammal associations for all similarity learners in all runs of our meta-ensemble (GBM). X-axes show the range of values of our similarity learners (0 to 1). Y-axes show the effect on the probability of coronavirus-mammal association (0 to 1) from that learner. Individual lines show the partial dependence per each run of the ensemble. The smoothed line (smoothed conditional means) indicates the overall trend of partial dependence between our response variable and the learner. Partial dependence measures the response for an individual variable in a machine-learning model (here GBM), while holding all other variable constant. Partial dependence plots visualise the non-linear relationships between each similarity-learner in our meta-ensemble and the response variable (whether a given coronavirus could potentially be found in a focal mammalian host).

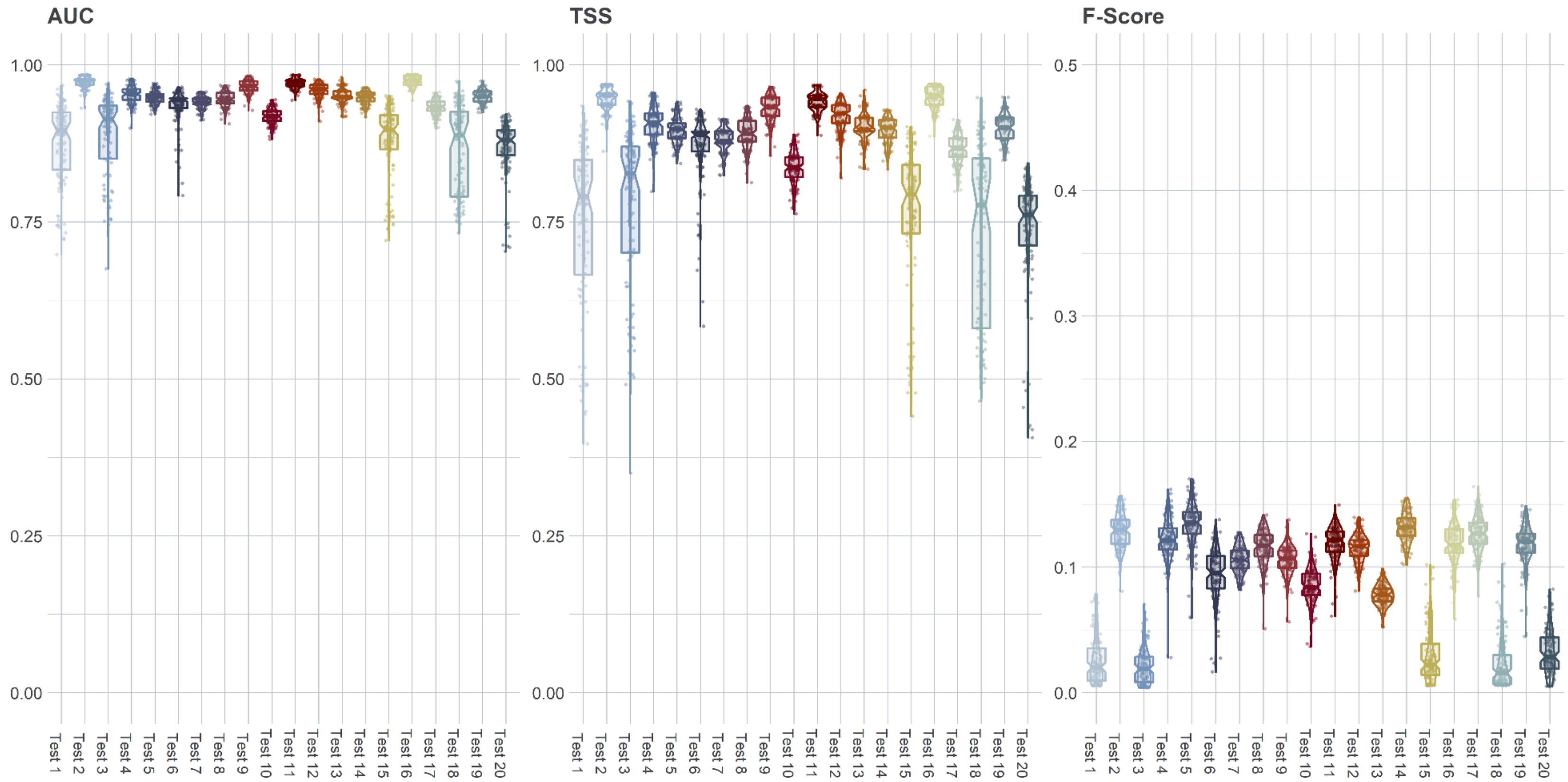

Supplementary Figure 7. Performance assessment over the held-out test sets (n =20) at 0.5 probability cut. Boxplots indicate median (center), the 25th and 75th percentiles (bounds of box) and minimum and maximum (whiskers). Violin plots show the kernel probability density of the data at different values. Points represent results from individual runs (100 runs per test). Boxplots, violin plots and points are coloured by test number. In each test we created a held-out test comprising 15% of all data (including 15% of all observed associations, and 15% of all unknown associations). Our learners were trained with the remainder 85% of the observed (and unknown) associations, and our meta-ensemble was then trained with predictions of these learners (10-fold cross validation, 100 repeats). Performance metrics were then computed against the held-out test set and reported here.

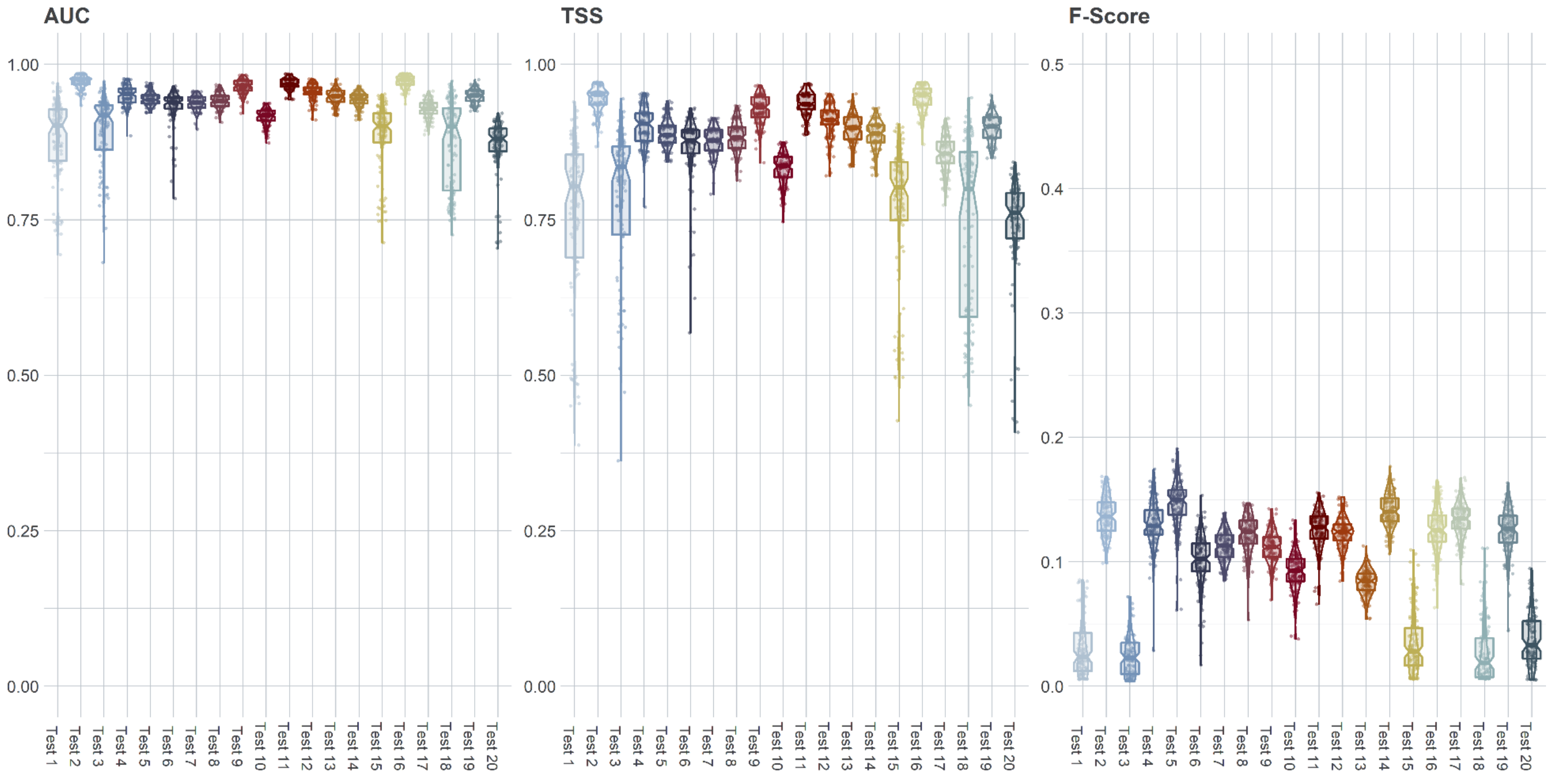

Supplementary Figure 8. Performance assessment over the held-out test sets (n =20) at 0.75 probability cut-off. Boxplots indicate median (center), the 25th and 75th percentiles (bounds of box) and minimum and maximum (whiskers). Violin plots show the kernel probability density of the data at different values. Points represent results from individual runs (100 runs per test). Boxplots, violin plots and points are coloured by test number. In each test we created a held-out test comprising 15% of all data (including 15% of all observed associations, and 15% of all unknown associations). Our learners were trained with the remainder 85% of the observed (and unknown) associations, and our meta-ensemble was then trained with predictions of these learners (10-fold cross validation, 100 repeats). Performance metrics were then computed against the held-out test set and reported here.

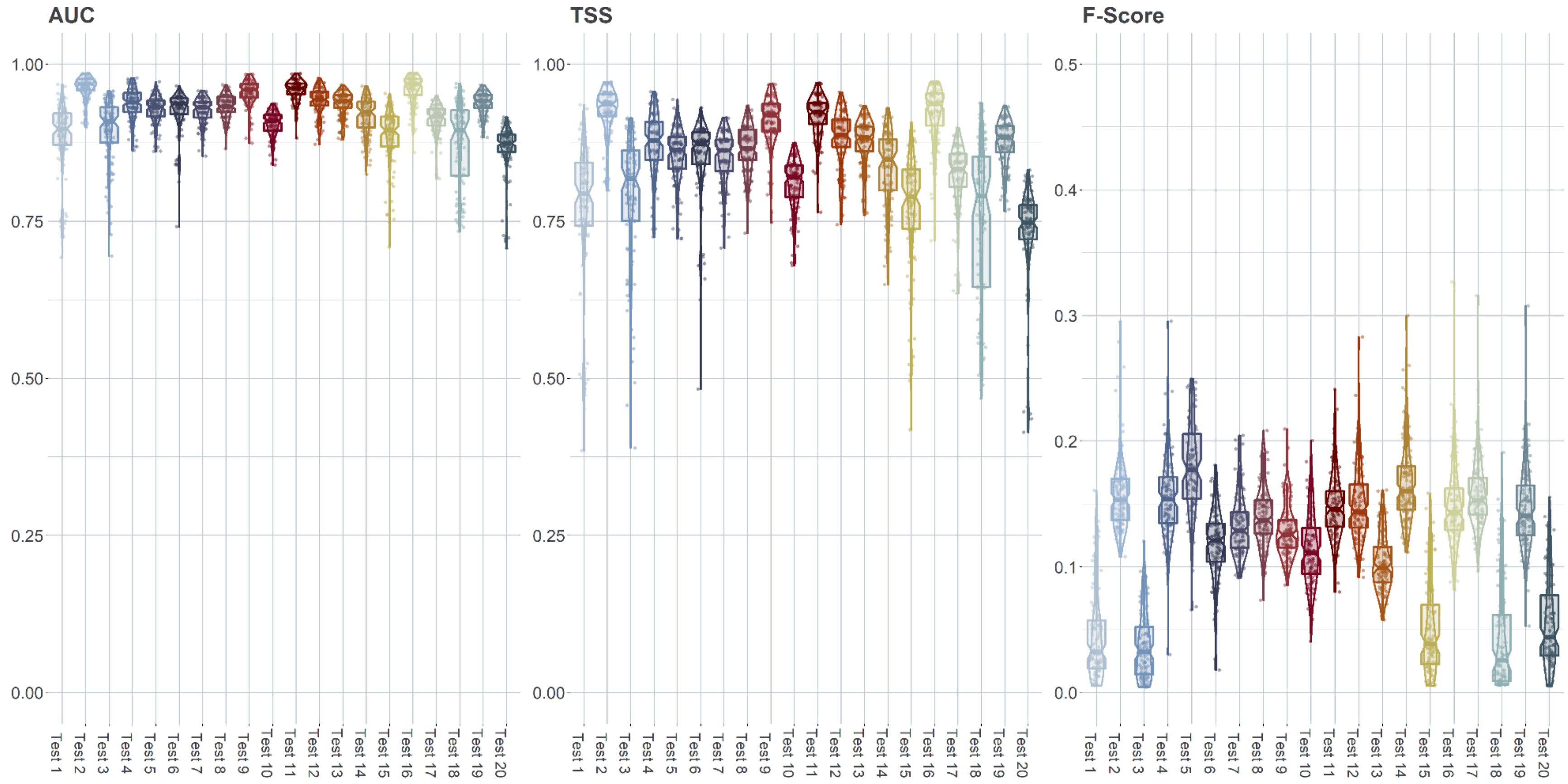

Supplementary Figure 9. Performance assessment over the held-out test sets (n =20) at 0.9821 probability cut-off. Boxplots indicate median (center), the 25th and 75th percentiles (bounds of box) and minimum and maximum (whiskers). Violin plots show the kernel probability density of the data at different values. Points represent results from individual runs (100 runs per test). Boxplots, violin plots and points are coloured by test number. In each test we created a held-out test comprising 15% of all data (including 15% of all observed associations, and 15% of all unknown associations). Our learners were trained with the remainder 85% of the observed (and unknown) associations, and our meta-ensemble was then trained with predictions of these learners (10-fold cross validation, 100 repeats). Performance metrics were then computed against the held-out test set and reported here.

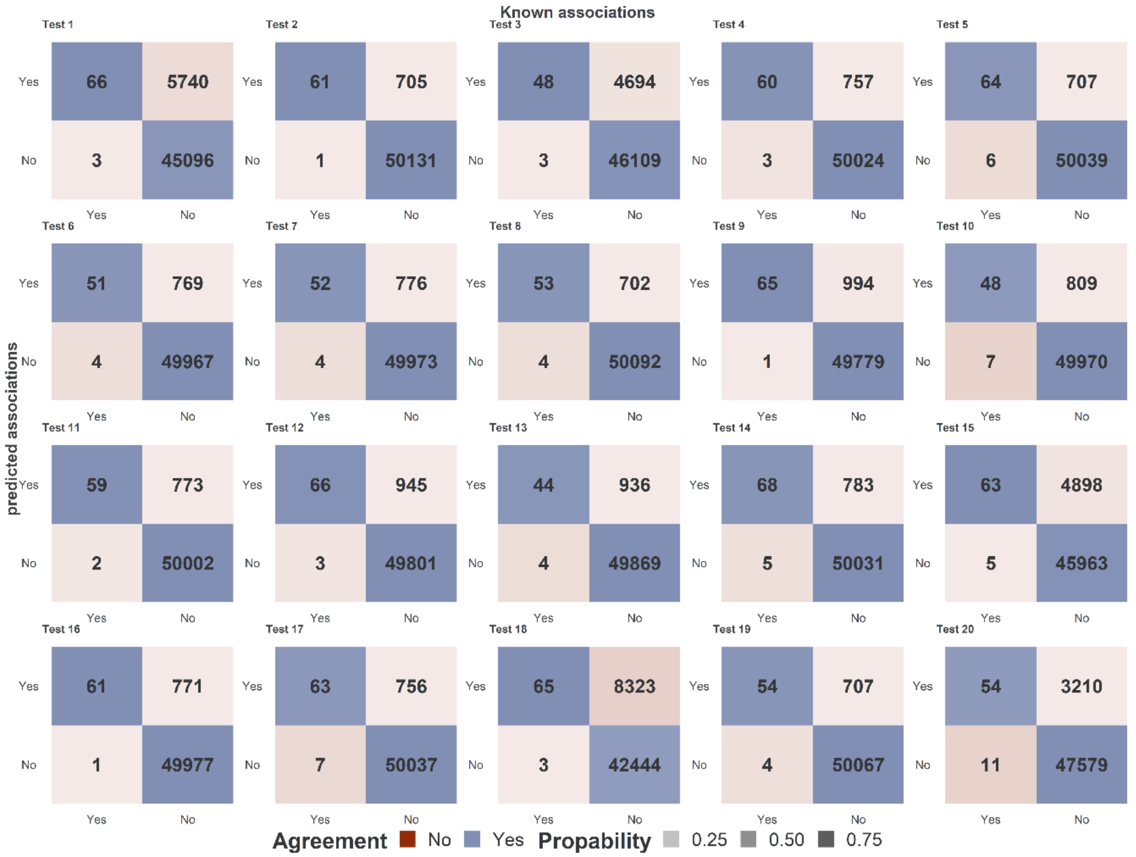

Supplementary Figure 10. Confusion matrices produced over the held-out test sets (n =20) at 0.5 probability cut-off. Confusion matrices were generated by taking the mean probability (across 100 runs) of our GBM meta-ensemble, predicted values>0.5 where considered positive (Yes), and those <=0.5 were considered negative (No). Colours in the above matrices indicate agreement between the predicted and the known associations (blue), or no agreement (blue). Transparency (alpha) indicates probability of agreement (the more times the two sets agreed, in relation to the total space (of yes or no) the more opaque the matrix cell.

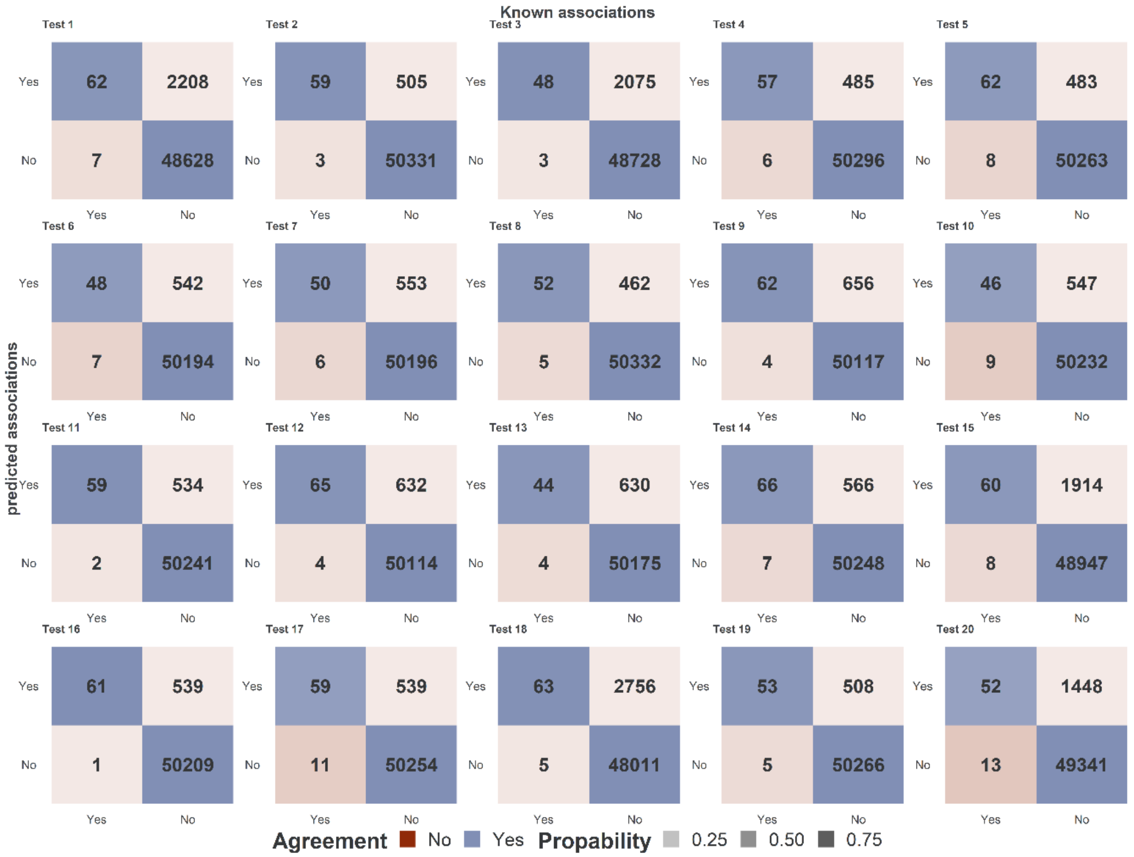

Supplementary Figure 11. Confusion matrices produced over the held-out test sets (n =20) at 0.75 probability cut-off. Confusion matrices were generated by taking the mean probability (across 100 runs) of our GBM meta-ensemble, predicted values>0.75 where considered positive (Yes), and those <=0.75 were considered negative (No). Colours in the above matrices indicate agreement between the predicted and the known associations (blue), or no agreement (blue). Transparency (alpha) indicates probability of agreement (the more times the two sets agreed, in relation to the total space (of yes or no) the more opaque the matrix cell.

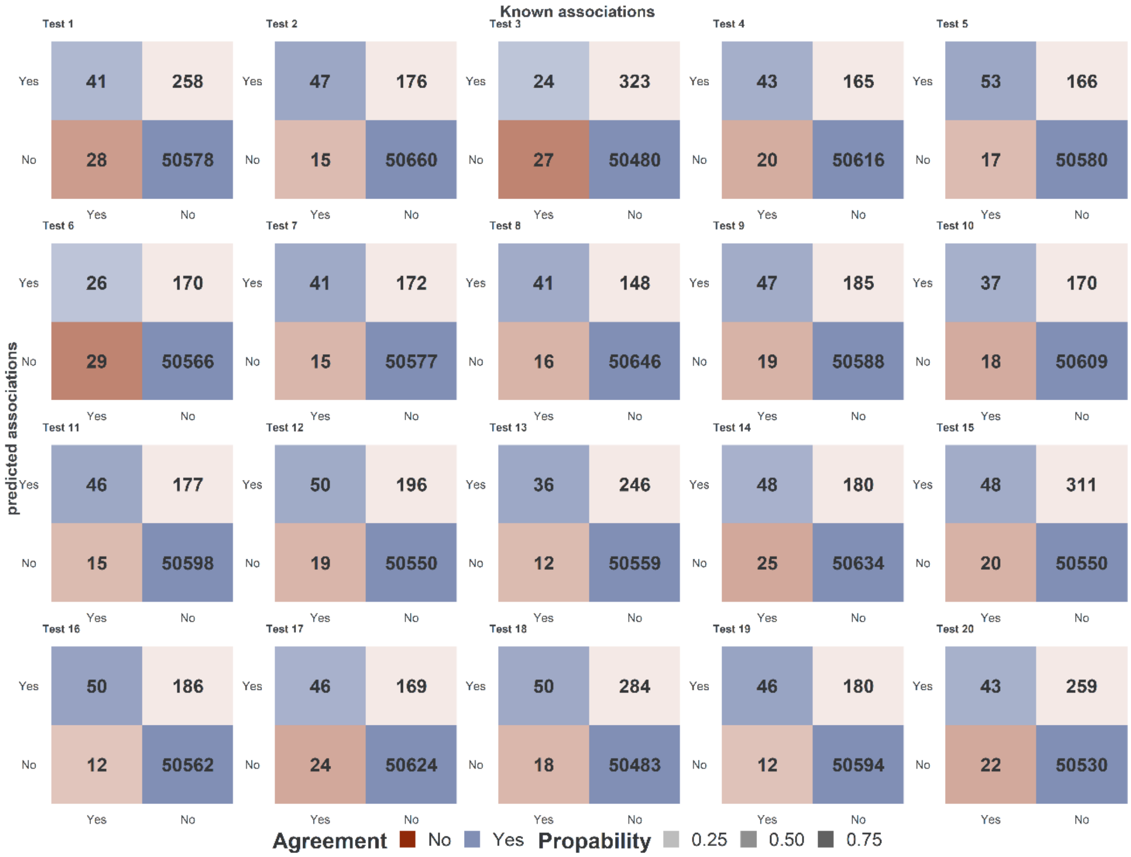

Supplementary Figure 12. Confusion matrices produced over the held-out test sets (n =20) at 0.9821 probability cut-off. Confusion matrices were generated by taking the mean probability (across 100 runs) of our GBM meta-ensemble, predicted values>=0.9821 where considered positive (Yes), and those <0.9 were considered negative (No). Colours in the above matrices indicate agreement between the predicted and the known associations (blue), or no agreement (blue). Transparency (alpha) indicates probability of agreement (the more times the two sets agreed, in relation to the total space (of yes or no) the more opaque the matrix cell.

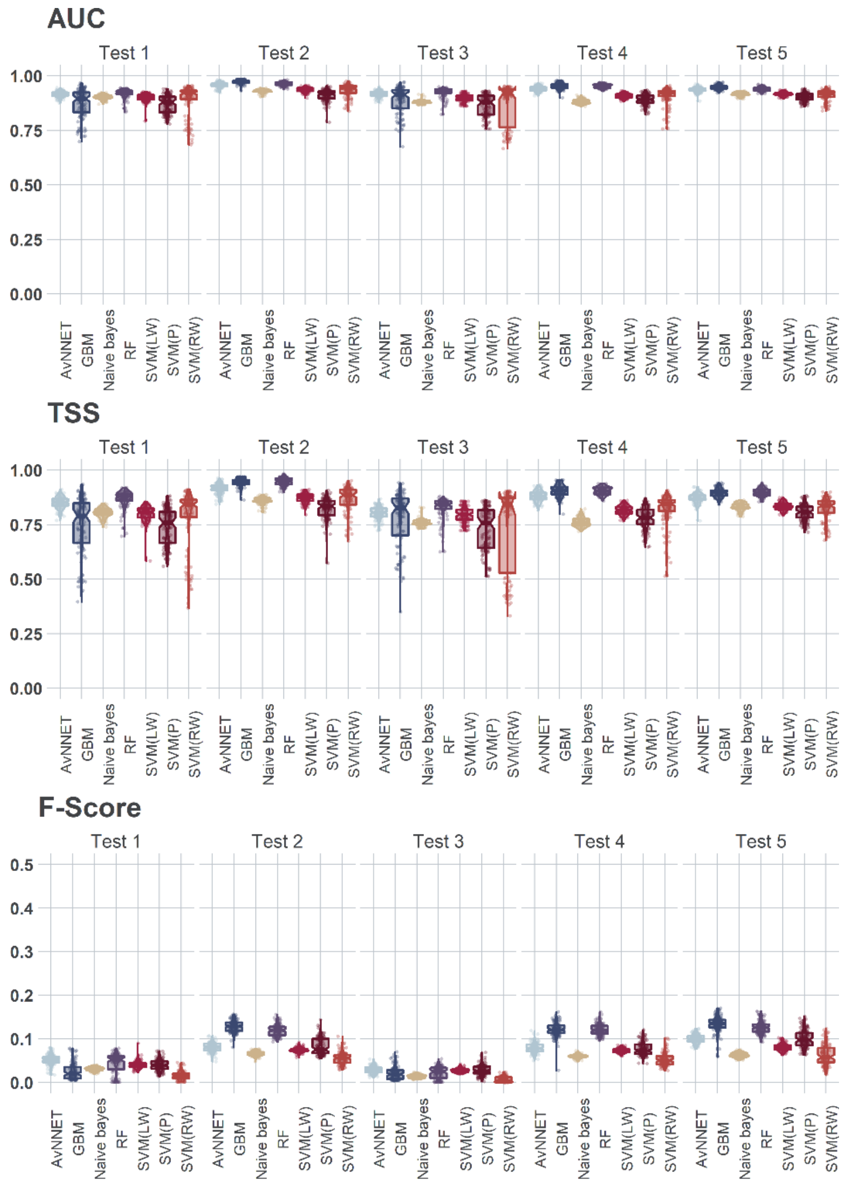

Supplementary Figure 13. Comparison of performance metrics of 7 classification algorithms over the held-out test sets (n = 5) at 0.5 probability cut-off. Notched boxplots indicate median (center), the 25th and 75th percentiles (bounds of box), and minimum and maximum (whiskers). Points represent results from individual runs (100 runs per test, and per algorithm). Boxplots and points are coloured by algorithm. We trained 7 classification algorithms: Model Averaged Neural Network (avNNet), Stochastic Gradient Boosting (GBM), Random Forest (RF), Support Vector Machines with radial basis kernel and class weights (SVM-RW), Linear SVM with Class Weights (SVM-LW), SVM with Polynomial Kernel (SVM-P), and Naive Bayes. These algorithms were selected due to their robustness, scalability, availability, and over-all performance. All models were trained and tested via caret R package. While no one algorithm performed best across all five tests, GBM performed well across the 5 tests, thus it was selected to perform the stacking of our similarity-based learners.

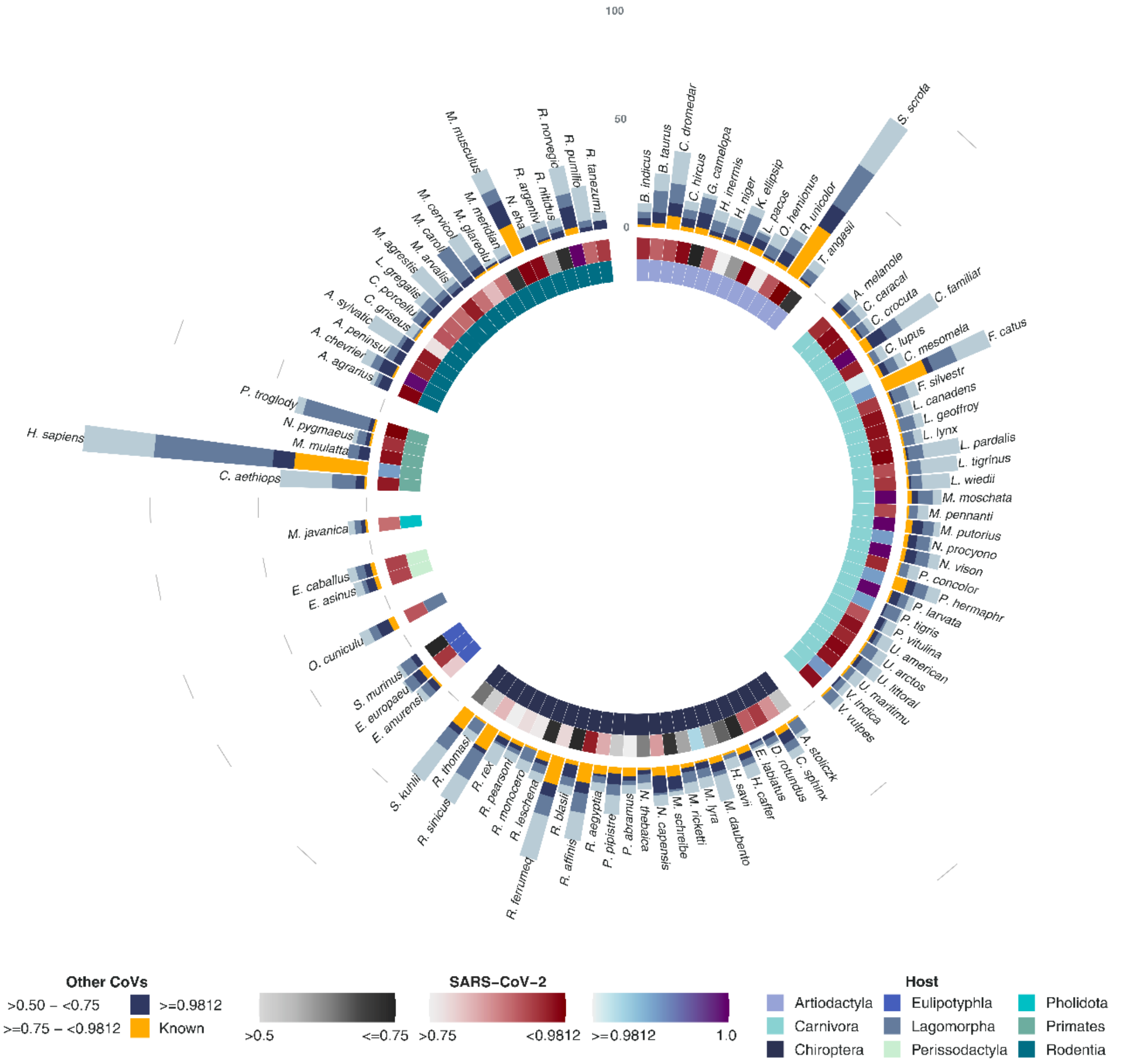

Supplementary Figure 14. Model predictions for potential hosts of SARS-Cov-2 – including humans and lab rodents. Predicted hosts are grouped by order (inner circle). Middle circle presents probability of association between host and SARS-CoV-2 (Grey scale indicates predicted associations with probability in range >0.5 - ≤0.75. Red scale indicates predicted associations with probability in range >0.75 - <0.9821. Blue to purple scale present indicates associations with probability ≥0.9821). Yellow bars represent number of coronaviruses (species or strains) observed to be found in each host. Blue stacked bars represent other coronaviruses predicted to be found in each host by our model. Predicted coronaviruses per host are grouped by prediction probability into three categories (from inside to outside): ≥0.9, >0.75 - <0.9821, and >0.5 - ≤0.75.

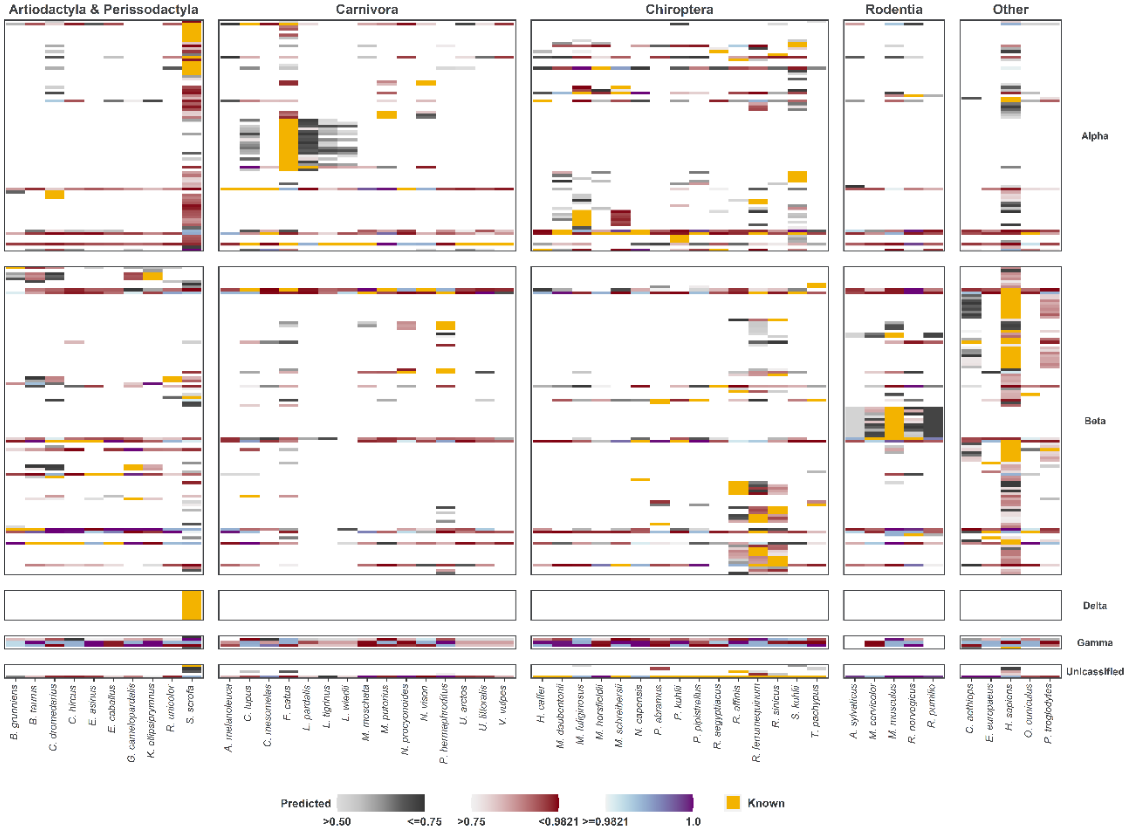

Supplementary Figure 15. Observed and predicted mammalian hosts for coronaviruses Including humans and lab rodents. Columns present mammalian hosts in four categories: Artiodactyla & Perissodactyla (top 10 hosts by number of predicted coronaviruses that could be found in each host); Carnivora (top 15 hosts), Chiroptera (top 15 hosts, each predicted to host 50 or more coronavirus species or strain), and others (top 5). Rows present viruses ordered into five taxonomic groups: Alphacoronaviruses, Betacoronaviruses, Deltacoronaviruses, Gammacoronaviruses and unclassified Coronavirinae. Yellow cells represent observed associations between the host and the coronavirus. Grey/red/blue cells indicate the probability of predicted associations in three increasing probability ranges. White cells indicate no known or predicted association between host and virus (beneath cut-off probability of 0.5).

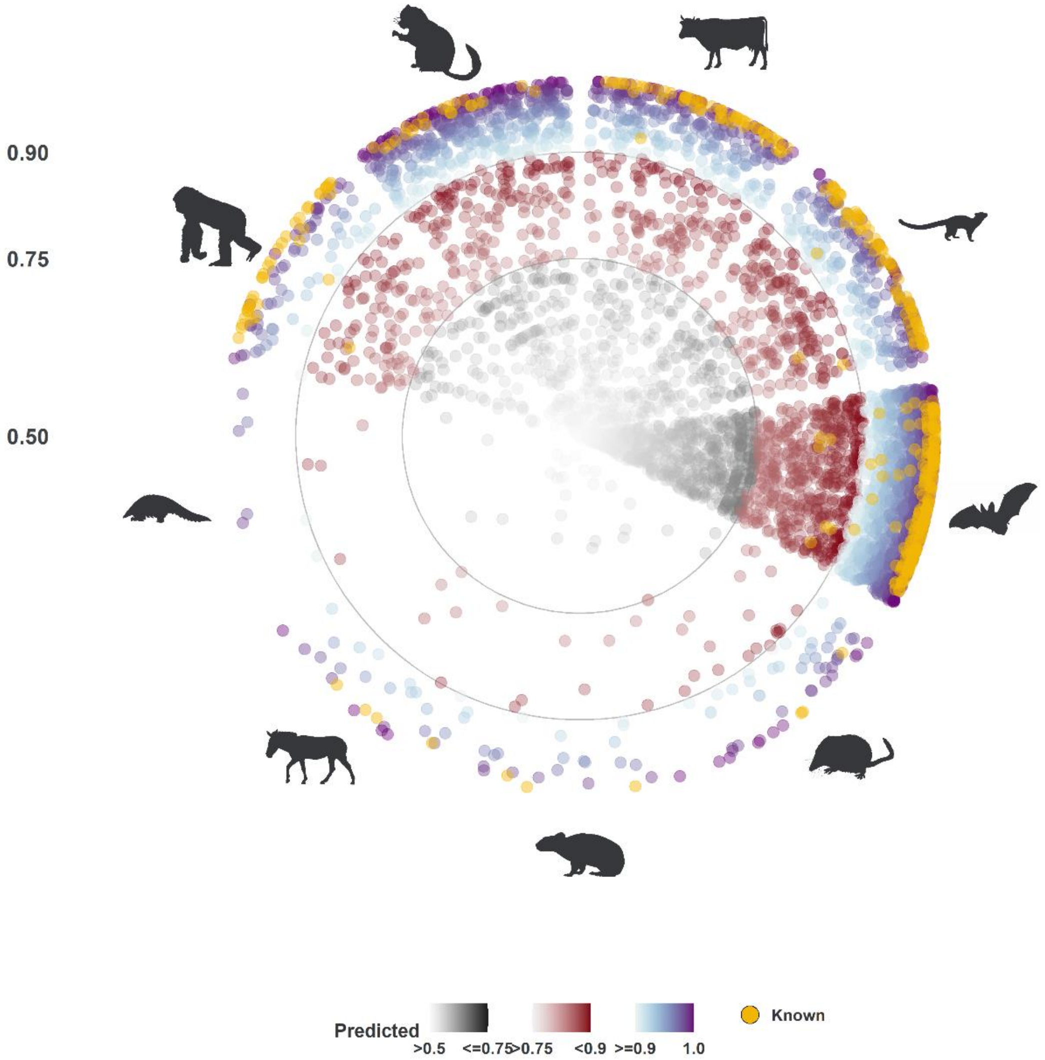

Supplementary Figure 16. Final model predictions and observed coronavirus-mammalian associations. Yellow circles present observed associations (known) between coronaviruses and mammalian hosts. Grey circles indicate associations predicted by the final model with probability >0.5 and <0.7. Red circles indicate associations predicted with probability <0.7 and <0.9. Blue-purple circles indicate associations predicted with probability ≥0.9. Y-axis represent the probability produced by the final model (trained with all available data, with 10-fold cross validation) – ranging from 0 to 1. X-axis represent the order of the mammalian host, as follows (clockwise): Artiodactyla, Carnivora, Chiroptera, Eulipotyphla, Lagomorpha, Perissodactyla, Pholidota, Primates, and Rodentia. Mammalian order silhouettes were obtained from phylopic.org. The percent of observed associations (known to exist between the focal mammal and the focal coronavirus) were predicted by the final model (trained with all available data) were as follows: 95.25% were predicted with probability cut-off≥0.9, whereas 97.37% were predicted with cut-offs: >0.75, and >0.5.

Supplementary references

1. Olival, K. J. *et al.* Host and viral traits predict zoonotic spillover from mammals. *Nature* **546**, 646–650 (2017). doi:10.1038/nature22975.
2. Guth, S., Visher, E., Boots, M. & Brook, C. E. Host phylogenetic distance drives trends in virus virulence and transmissibility across the animal–human interface. *Philos. Trans. R. Soc. B Biol. Sci.* **374**, 20190296 (2019).
3. Longdon, B., Brockhurst, M. A., Russell, C. A., Welch, J. J. & Jiggins, F. M. The Evolution and Genetics of Virus Host Shifts. *PLoS Pathog.* **10**, e1004395 (2014).
4. Fritz, S. A., Bininda-Emonds, O. R. P. & Purvis, A. Geographical variation in predictors of mammalian extinction risk: big is bad, but only in the tropics. *Ecol. Lett.* **12**, 538–549 (2009). doi: 10.1111/j.1461-0248.2009.01307.x
5. Jones, K. E. *et al.* PanTHERIA: a species-level database of life history, ecology, and geography of extant and recently extinct mammals. *Ecology* **90**, 2648–2648 (2009). doi: 10.1890/08-1494.1.
6. Gnanadesikan, G. E., Pearse, W. D. & Shaw, A. K. Evolution of mammalian migrations for refuge, breeding, and food. *Ecol. Evol.* **7**, 5891–5900 (2017). doi: 10.1002/ece3.3120.
7. Wilman, H. *et al.* EltonTraits 1.0: Species-level foraging attributes of the world's birds and mammals. *Ecology* **95**, 2027–2027 (2014). doi: 10.1890/13-1917.1.
8. IUCN 2020. The IUCN Red List of Threatened Species. Version 2020-3. Available at: http://www.iucnredlist.org. doi: 10.2305/IUCN.UK.2019-2.RLTS.T61138A148835518.en.
9. DE MAGALHÃES, J. P. & COSTA, J. A database of vertebrate longevity records and their relation to other life-history traits. *J. Evol. Biol.* **22**, 1770–1774 (2009).doi: 10.1111/j.1420-9101.2009.01783.x
10. Gower, J. C. A General Coefficient of Similarity and Some of Its Properties. *Biometrics* **27**, 857 (1971).
11. Pavoine, S., Vallet, J., Dufour, A.-B., Gachet, S. & Daniel, H. On the challenge of treating various types of variables: application for improving the measurement of functional diversity. *Oikos* **118**, 391–402 (2009).
12. McIntyre, K. M. *et al.* Systematic Assessment of the Climate Sensitivity of Important Human and Domestic Animals Pathogens in Europe. *Sci. Rep.* **7**, 7134 (2017).
13. Hay, S. I. *et al.* Global mapping of infectious disease. *Philos. Trans. R. Soc. Lond. B. Biol. Sci.* **368**, 20120250 (2013).
14. Anyamba, A. *et al.* Global Disease Outbreaks Associated with the 2015–2016 El Niño Event. *Sci. Rep.* **9**, 1930 (2019).
15. Karesh, W. B. *et al.* Ecology of zoonoses: natural and unnatural histories. *Lancet* **380**, 1936–1945 (2012).
16. Hassell, J. M., Begon, M., Ward, M. J. & Fèvre, E. M. Urbanization and Disease Emergence: Dynamics at the Wildlife-Livestock-Human Interface. *Trends Ecol. Evol.* **32**, 55–67 (2017).
17. Gilbert, M. *et al.* Global distribution data for cattle, buffaloes, horses, sheep, goats, pigs, chickens and ducks in 2010. *Sci. Data* **5**, 180227 (2018). doi: 10.1038/sdata.2018.227.
18. University, C. for I. E. S. I. N.-C.-C. Gridded Population of the World, Version 4 (GPWv4): Population Density Adjusted to Match 2015 Revision UN WPP Country Totals, Revision 10. (2017).doi: 10.7927/H49C6VHW.
19. Harris, I., Jones, P. D., Osborn, T. J. & Lister, D. H. Updated high-resolution grids of monthly climatic observations - the CRU TS3.10 Dataset. *Int. J. Climatol.* **34**, 623–642 (2014).doi: 10.1002/joc.3711.
20. IUCN, I. U. for C. of N.- & University, C. for I. E. S. I. N.-C.-C. Gridded Species Distribution: Global Mammal Richness Grids, 2015 Release. (2015).doi: 10.7927/H4N014G5.
21. Hopkins, M. E. & Nunn, C. L. A global gap analysis of infectious agents in wild primates. *Divers. Distrib.* **13**, 561–572 (2007).doi: 10.1111/j.1472-4642.2007.00364.x.
22. Lloyd-Smith, J. O. *et al.* Epidemic Dynamics at the Human-Animal Interface. *Science (80-.).* **326**, 1362–1367 (2009).

23. Jenkins, C. N., Pimm, S. L. & Joppa, L. N. Global patterns of terrestrial vertebrate diversity and conservation. *Proc. Natl. Acad. Sci. U. S. A.* **110**, E2602-10 (2013).
24. Lloyd-Smith, J. O. *et al.* Epidemic dynamics at the human-animal interface. *Science* **326**, 1362–7 (2009).
25. Jones, B. A. *et al.* Zoonosis emergence linked to agricultural intensification and environmental change. *Proc. Natl. Acad. Sci.* **110**, 8399–8404 (2013).
26. Weaver, S. C. Urbanization and geographic expansion of zoonotic arboviral diseases: mechanisms and potential strategies for prevention. *Trends Microbiol.* **21**, 360–3 (2013).
27. Eskew, E. A. & Olival, K. J. De-urbanization and Zoonotic Disease Risk. *Ecohealth* (2018). doi:10.1007/s10393-018-1359-9
28. Jones, K. E. *et al.* Global trends in emerging infectious diseases. *Nature* **451**, 990–993 (2008).
29. Dunn, R. R., Davies, T. J., Harris, N. C. & Gavin, M. C. Global drivers of human pathogen richness and prevalence. *Proc. R. Soc. B Biol. Sci.* **277**, 2587–2595 (2010).
30. Wolfe, N. D., Dunavan, C. P. & Diamond, J. Origins of major human infectious diseases. *Nature* **447**, 279–283 (2007).
31. Allen, T. *et al.* Global hotspots and correlates of emerging zoonotic diseases. *Nat. Commun.* **8**, 1124 (2017).
32. Wang, B. *et al.* Similarity network fusion for aggregating data types on a genomic scale. *Nat. Methods* **11**, 333–337 (2014).
33. Perozzi, B., Al-Rfou, R. & Skiena, S. DeepWalk: Online Learning of Social Representations. *Proc. ACM SIGKDD Int. Conf. Knowl. Discov. Data Min.* 701–710 (2014). doi:10.1145/2623330.2623732
34. Mikolov, T., Chen, K., Corrado, G. & Dean, J. Efficient estimation of word representations in vector space. in *1st International Conference on Learning Representations, ICLR 2013 - Workshop Track Proceedings* (International Conference on Learning Representations, ICLR, 2013).
35. Mnih, A. & Hinton, G. *A Scalable Hierarchical Distributed Language Model*. (2009).
36. Ulrich, W. Ecological interaction networks: prospects and pitfalls. *Ecol. Quest.* **11**, 17 (2009).
37. Stone, L. & Roberts, A. Competitive exclusion, or species aggregation? - An aid in deciding. *Oecologia* **91**, 419–424 (1992).
38. Connor, E. F., Collins, M. D. & Simberloff, D. The checkered history of checkerboard distributions. *Ecology* **94**, 2403–2414 (2013).
39. Almeida-Neto, M., Guimarães, P., Guimarães, P. R., Loyola, R. D. & Ulrich, W. A consistent metric for nestedness analysis in ecological systems: Reconciling concept and measurement. *Oikos* **117**, 1227–1239 (2008).
40. Staniczenko, P. P. A., Kopp, J. C. & Allesina, S. The ghost of nestedness in ecological networks. *Nat. Commun.* **4**, 1–6 (2013).
41. Thébault, E. & Fontaine, C. Stability of ecological communities and the architecture of mutualistic and trophic networks. *Science (80-.).* **329**, 853–856 (2010).
42. Dormann, C. F., Gruber, B. & Fründ, J. *Introducing the bipartite Package: Analysing Ecological Networks*. **8**, (2008).
