# Supplementary material for: Predicting mammalian hosts in which novel coronaviruses can be generated
Source: Nat Commun. 2021 Feb 16;12:780. doi: 10.1038/s41467-021-21034-5 (PMC7887240; doi:10.1038/s41467-021-21034-5)
Supplement: Supplementary file 3 — Description of Additional Supplementary Files [file 41467_2021_21034_MOESM3_ESM.pdf]

Description of Additional Supplementary Files

File Name: Supplementary Data 1
Description: Predicted non-human mammalian hosts of SARS-CoV-2, excluding labrodents.

File Name: Supplementary Data 2
Description: Observed and predicted mammalian host-range (1 or more) of coronaviruses (species and strains). Values in brackets represent +/- SD from ensemble mean.

File Name: Supplementary Data 3
Description: Predicted number of coronaviruses (1 or more, species or strain) per nonhuman mammalian species. Values in bracket represent +/- SD from ensemble mean.

File Name: Supplementary Data 4
Description: Observed and predicted number of coronaviruses for 50 mammalian species (as presented in Figure 2), excluding humans and lab rodents. Values in bracket represent +/- SD from ensemble mean.

File Name: Supplementary Data 5
Description: Predicted coronaviruses that could be found in humans. Minimum probability cut-off>0.5.

File Name: Supplementary Data 6
Description: Potential recombination mammalian hosts of both: SARS-CoV-2 and MERSCoV (middle east respiratory syndrome-related coronavirus). Probability cut-off>0.5.

File Name: Supplementary Data 7
Description: NCBI Accession and GI numbers of the sequences used in the analyses.
