# Supplementary material for: Predicting mammalian hosts in which novel coronaviruses can be generated
Source: Nat Commun. 2021 Feb 16;12:780. doi: 10.1038/s41467-021-21034-5 (PMC7887240; doi:10.1038/s41467-021-21034-5)
Supplement: Supplementary file 11 — Reporting Summary [file 41467_2021_21034_MOESM11_ESM.pdf]

nature research

Corresponding author(s): Maya Wardeh, Marcus Blagrove

Last updated by author(s): Jan 7, 2021

Reporting Summary

Nature Research wishes to improve the reproducibility of the work that we publish. This form provides structure for consistency and transparency in reporting. For further information on Nature Research policies, see our Editorial Policies and the Editorial Policy Checklist.

Statistics

For all statistical analyses, confirm that the following items are present in the figure legend, table legend, main text, or Methods section.

n/a	Confirmed	
☐	☒	The exact sample size (*n*) for each experimental group/condition, given as a discrete number and unit of measurement
☐	☒	A statement on whether measurements were taken from distinct samples or whether the same sample was measured repeatedly
☐	☒	The statistical test(s) used AND whether they are one- or two-sided<br>*Only common tests should be described solely by name; describe more complex techniques in the Methods section.*
☐	☒	A description of all covariates tested
☐	☒	A description of any assumptions or corrections, such as tests of normality and adjustment for multiple comparisons
☒	☐	A full description of the statistical parameters including central tendency (e.g. means) or other basic estimates (e.g. regression coefficient) AND variation (e.g. standard deviation) or associated estimates of uncertainty (e.g. confidence intervals)
☒	☐	For null hypothesis testing, the test statistic (e.g. *F*, *t*, *r*) with confidence intervals, effect sizes, degrees of freedom and *P* value noted<br>*Give P values as exact values whenever suitable.*
☒	☐	For Bayesian analysis, information on the choice of priors and Markov chain Monte Carlo settings
☐	☒	For hierarchical and complex designs, identification of the appropriate level for tests and full reporting of outcomes
☒	☐	Estimates of effect sizes (e.g. Cohen's *d*, Pearson's *r*), indicating how they were calculated

Our web collection on statistics for biologists contains articles on many of the points above.

Software and code

Policy information about availability of computer code

Data collection	No specialised software was used in data collection.
Data analysis	R version 3.6.2 was used for data analysis. All algorithms used to train stacking (meta-)ensembles (GBM, random forest (ranger), AvNNEt, and support vector machines) were trained using the R package caret (version 6.0.85). Python version 3.7.3 (PyCharm) and deepwalk 1.0.3 were used to run the DeepWalk algorithm (including skip-grams). All codes used in our analyses are made available via figshare: (https://doi.org/10.6084/m9.figshare.13110896).

For manuscripts utilizing custom algorithms or software that are central to the research but not yet described in published literature, software must be made available to editors and reviewers. We strongly encourage code deposition in a community repository (e.g. GitHub). See the Nature Research guidelines for submitting code & software for further information.

Data

Policy information about availability of data

All manuscripts must include a data availability statement. This statement should provide the following information, where applicable:
- Accession codes, unique identifiers, or web links for publicly available datasets
- A list of figures that have associated raw data
- A description of any restrictions on data availability

Genomic sequences of coronaviruses were obtained from NCBI GenBank, accession codes are listed in Supplementary Data 7. Coronaviruses-hosts associations were obtained from the ENHanCEd Infectious Diseases Database (EID2: https://eid2.liverpool.ac.uk/). Mammalian and geospatial data were obtained from open-access data sources. These sources are listed in detail, and their DOIs are provided in the Supplementary Information file. Data used can be found here: https://doi.org/10.6084/m9.figshare.13110896, with the exception of mammalian presence shapefiles and raw climate data (due to their large size) - these data can be obtained from the authors or directly from the sources listed in the Supplementary Information file.

Field-specific reporting

Please select the one below that is the best fit for your research. If you are not sure, read the appropriate sections before making your selection.

☐ Life sciences ☐ Behavioural & social sciences ☒ Ecological, evolutionary & environmental sciences

For a reference copy of the document with all sections, see nature.com/documents/nr-reporting-summary-flat.pdf

Ecological, evolutionary & environmental sciences study design

All studies must disclose on these points even when the disclosure is negative.

Study description	Novel pathogenic coronaviruses – including SARS-CoV and SARS-CoV-2 – arise by homologous recombination in a host cell. This process requires a single host to be infected with more than one type of coronavirus, which recombine to form novel strains of virus. Identifying possible sources of novel coronaviruses requires identifying these hosts (termed recombination hosts). However, the majority of coronavirus-host interactions remain unknown, and therefore the vast majority of recombination hosts for coronaviruses cannot be identified. Here we deploy a meta-ensemble of similarity learners from three complementary perspectives: viral (genome), mammalian (ecology, distribution and phylogeny) and the network connecting coronaviruses and hosts to predict which mammals are hosts of multiple coronaviruses and thus can be considered recombination hosts in which novel coronaviruses could be generated.
Research sample	Documented associations between coronaviruses and their hosts were obtained from the Enhanced Infectious Diseases Database (EID2: https://eid2.liverpool.ac.uk/)(Wardeh et al, 2015). EID2 is the largest source of pathogen-host association, and is fully evidence based (PubMed publications and/or meta-data associated with genomes uploaded to NCBI Nucleotide database).
Sampling strategy	We used all mammalian species in which at least one coronavirus species or strain has been detected. We supplemented this set with all mammalian species within the genera of these mammalian hosts.
Data collection	No data were collected for this study.
Timing and spatial scale	No data were collected for this study.
Data exclusions	We excluded non-terrestrial mammalian species from our analyses due to non-availability of geo-spatial traits (e.g. land-cover type, climate data).
Reproducibility	Analyses are fully reproducible, determined by running the R & python scripts made available via figshare.
Randomization	No data were collected for this study.
Blinding	No data were collected for this study.

Did the study involve field work? ☐ Yes ☒ No

Reporting for specific materials, systems and methods

We require information from authors about some types of materials, experimental systems and methods used in many studies. Here, indicate whether each material, system or method listed is relevant to your study. If you are not sure if a list item applies to your research, read the appropriate section before selecting a response.

Materials & experimental systems

n/a	Involved in the study
☒	☐ Antibodies
☒	☐ Eukaryotic cell lines
☒	☐ Palaeontology and archaeology
☒	☐ Animals and other organisms
☒	☐ Human research participants
☒	☐ Clinical data
☒	☐ Dual use research of concern

Methods

n/a	Involved in the study
☒	☐ ChIP-seq
☒	☐ Flow cytometry
☒	☐ MRI-based neuroimaging
